# Pumpkin Skin Polysaccharide–Zn(II) Complex: Preparation, Characterization, and Suppression of Inflammation in Zebrafish

**DOI:** 10.3390/foods11172610

**Published:** 2022-08-28

**Authors:** Shujun Dong, Bin Zhang, Yue Ma, Hong Chang, Zhenjia Zheng, Xiaoyan Zhao

**Affiliations:** 1 Beijing Key Laboratory of Agricultural Products of Fruits and Vegetables Preservation and Processing, Key Laboratory of Vegetable Postharvest Processing, Ministry of Agriculture and Rural Affairs, Institute of Agri-food Processing and Nutrition, Beijing Academy of Agriculture and Forestry Sciences, Beijing 100097, China; 2 Key Laboratory of Food Processing Technology and Quality Control of Shandong Higher Education Institutes, College of Food Science and Engineering, Shandong Agricultural University, 61 Daizong Street, Tai’an 271018, China

**Keywords:** pumpkin skin polysaccharides, zinc complex, physicochemical property, anti-inflammatory activity

## Abstract

In this study, pumpkin (*Cucurbita moschata*) skin polysaccharide–zinc(II) (PSP−Zn) complex was successfully prepared. The structure and physicochemical properties of PSP and PSP−Zn were analyzed. The anti-inflammatory activity of PSP and PSP−Zn was investigated in zebrafish larvae induced by copper sulphate. PSP and PSP−Zn consisted of rhamnose, arabinose, galactose, glucose, and galacturonic acid. The molecular weight (Mw) of PSP and PSP−Zn were 3.034 × 10^6^ and 3.222 × 10^6^ Da, respectively. Fourier transform infrared spectrum (FT-IR) and circular dichroism (CD) analysis results suggested that the chemical modification of zinc might occur through hydroxyl groups of PSP. The PSP−Zn complex had lamellar texture, smooth surface morphology, and larger particle size. X-ray Diffraction (XRD) analysis revealed that both PSP and PSP−Zn were semi-crystalline substances. PSP−Zn solution showed superior stability in a weak acid and alkaline environment, especially at pH = 6.0. Moreover, PSP and PSP−Zn showed a good inhibitory effect on inflammation cells in zebrafish. Real-time quantitative polymerase chain reaction (RT-PCR) result suggested that the anti-inflammatory mechanism of PSP and PSP−Zn were through downregulation of the expression of nitric oxide synthase 2b (nos2b), inducible nitric oxide synthase (iNOS), interleukin-6 (IL-6), and nuclear factor-kappa B2 (NF-κB2). The present study indicated that PSP−Zn is expected to be a safe and efficient novel zinc supplement with anti-inflammatory activity.

## 1. Introduction

Zinc is one of the essential trace elements in the human body and has a variety of physiological functions. It plays a vital role in improving the immune function of the body, transporting the signal of in vivo cells, and regulating the activity of various hormones [1]. Deficiency of zinc leads to growth retardation, decreased immunity, and a higher risk of cardio-cerebrovascular disease and even cancer. Mild or moderate zinc deficiency is considered to affect people worldwide, especially children and pregnant women. However, inorganic zinc supplementations such as zinc chloride are suspected to exhibit low bioavailability, poor zinc absorption, and toxicity in the gastrointestinal tract [2,3].

Polysaccharides are a kind of polyhydroxy compound with negative charges, with stronger hydrogen atom or electron donation capacities. Therefore, they have great potential as metal ion organic ligands [4]. Biological and pharmacological studies have shown that polysaccharides possess a variety of bioactivities, including antioxidant, anti-inflammatory, immunomodulation, anti-tumor, and anti-diabetic activity, which were all shown to be related to their structural characteristics [5,6,7,8,9]. In recent years, many studies have investigated polysaccharide–metal complexes or chelates, including polysaccharide–iron, –copper, –calcium, and –zinc complexes. New biological zinc supplements have been shown to be highly bioactive, non-toxic, and harmless [3,10].

Pumpkin (*Cucurbita moschata*) belongs to the genus *Cucurbita* and family *Cucurbitaceae*, known as a nutritional food with a long history in oriental countries [11]. Previous reports have indicated that the major bioactive component in pumpkin was polysaccharides, extracted from pumpkin pulp and seeds [12,13]. Reportedly, the molecular weight, extraction method, monosaccharide composition, and glycosidic bond type of pumpkin polysaccharides are pivotal in their biological activity [14]. The thick layer of pumpkin skin is usually treated as a waste of processing, which accounts for about 10-40% of the total quantity of the pumpkin, resulting in a low comprehensive utilization rate and environmental pollution [15]. To the best of our knowledge, there has been no report on pumpkin skin polysaccharides at present, not to mention their application.

Zebrafish (*Danio rerio*) have emerged as an unprecedented tool for rapidly and economically investigating immune functions in recent years. As a vertebrate, zebrafish have the advantages of tiny body size, rapid growth, high fecundity, and rapid breeding. In addition, the transparency of zebrafish embryos and larvae enables real-time visualization of fluorescent proteins in vivo [16]. The innate immune system and neutrophils are developed as early as 3 days post fertilization (3 dpf) [17]. Moreover, various transgenic zebrafish lines have been successfully used for anti-inflammatory research. For example, Tg(mpx:EGFP) and Tg(Lyz:EGFP) zebrafish lines are commonly used for observing neutrophils in inflammatory areas in real time, as their neutrophil proteins express green fluorescence [18,19].

Inspired by the aforementioned results, the present study is aimed at preparing the pumpkin skin polysaccharide–zinc (PSP−Zn) complex and investigating its physicochemical characteristics and anti-inflammatory activity in vivo. In this study, the physicochemical characteristics of PSP and PSP−Zn were assayed by HPLC and HPAEC methods; the structures of PSP and PSP−Zn were characterized by FTIR, CD, XRD, TGA, and SEM. Moreover, the anti-inflammatory activity was evaluated in zebrafish (Tg-Lyz:EGFP). This study can provide new ideas for the development of novel zinc supplements and the exploitation of pumpkin resources.

## 2. Materials and Methods

### 2.1. Materials

Pumpkin (commercial cultivar: Aibisi) skin was provided by Mengyu Food Co., Ltd. (Yantai, China). ZnSO_4_·7H_2_O of analytical grade was purchased from Kaitong Chemical Reagent Co., Ltd. (Tianjin, China). Monosaccharides standards (rhamnose, arabinose, galactose, glucose, mannose, xylose, fructose, galacturonic acid, and glucuronic acid) and dextran were purchased from Shanghai Yuanye Biological Technology Co. Ltd. (Shanghai, China). Trifluoroacetic acid (TFA), CuSO_4_, and ibuprofen were purchased from Sigma-Aldrich (St. Louis, MO, USA). RNA Extraction Kit (RC101) and SYBR (Ace qPCR) were purchased from Vazyme Co., Ltd. (Nanjing, China).

### 2.2. Extraction of PSP and Preparation of PSP−Zn

The PSP extraction procedure was based on the method of Wang et al. (2017) with some modification [12]. Briefly, the pumpkin skin powder was obtained after freeze-drying. The PSP was extracted with distilled water (1:12, *v/w*) at 80 °C for 2 h, followed by centrifuging the homogenate at 5500× *g* for 10 min to obtain crude PSP. A savage reagent (chloroform:butyl alcohol = 4:1, *v/v*) was added to the crude PSP to remove protein. After that, anhydrous ethanol was added to the crude PSP (1:4, *v/v*) to precipitate the polysaccharides. The PSP was lyophilized by a freeze drier.

The preparation procedure of PSP−Zn was based on a previously study [3]. Firstly, PSP (10 g/L) was mixed with zinc sulfate solution (0.56 g/L; 1:1, *v/v*). The pH of the reaction solution was adjusted to 9.0 with 0.5 mol/L NaOH. The reaction was conducted in a water bath at 50 °C for 104 min, and alcohol precipitation centrifugation was performed. The product was then dialyzed with deionized water for 48 h, changing the water every 12 h. Finally, PSP−Zn was obtained by freeze-drying. The zinc contents were analyzed by inductively coupled plasma mass spectrometry (ICP-MS). The zinc content of PSP−Zn was 23.17 mg/g.

### 2.3. Structural Characterization of PSP and PSP−Zn

#### 2.3.1. Determination of Molecular Weight

The molecular weights (Mw) of PSP and PSP−Zn were determined using a HPLC-LC-20A/RID system (Shimadzu, Japan) containing a Shodex SB-806 HQ gel column. The samples were analyzed as follows: mobile phase of 0.1 mol/L NaNO_3_, flow rate of 0.5 mL/min, column temperature of 40 °C, sample injection volume of 200 μL. A set of different Mw dextran standards were used as standards to obtain a calibration curve. The molecular weights of polysaccharides were estimated with the natural logarithm of the relative Mw plotted and the retention time.

#### 2.3.2. Monosaccharide Composition Analysis

The monosaccharide composition analysis was determined by high-performance anion-exchange chromatography with pulsed amperometric detection (HPAEC-PAD, Thermo Fisher ICS-5000+, Thermo Fisher, Waltham, MA, USA), adopting the method described by a previous report with slight modifications [20]. Briefly, 10 mg of PSP and PSP−Zn were hydrolyzed using 2 mL of 2.0 mol/L TFA at 80 °C for 5 h. The hydrolyzed product was dissolved in 20 mL water, followed by removing the residual TFA with a rotary evaporator at reduced pressure at 60 °C. For accurate qualitative and quantitative analysis of monosaccharides, nine monosaccharide standards were chosen as the external standards. All samples were clarificated by Supelclean™ENVI-18 SPE tube (Merk, MO, USA) and 0.22 μm millipore filter before analysis. The resulting samples were then analyzed using a Dionex™AminoPac™PA10 IC column (3 × 250 mm, Dionex, CA, USA,) with a mobile phase of 0.20 mol/L NaOH, a flow rate of 0.25 mL/min, and a column temperature of 30 °C. In addition, the m-hydroxydiphenyl method was used for determining the uronic acid content as described by Kinttner and Van Buren [21]. Next, 1 mL of the resulting sample was mixed with 6-fold volume of borax/sulfuric acid solution (0.478/100, *w*/*v*), which was then incubated at 100 °C for 5 min. After that, 0.1 mL of m- m-hydroxydiphenyl (1.5 mg/mL) was added to the mixture, with a reaction time of 20 min. The uronic acids were determined with a UV-2450 pectrophotometer (Shimadzu, Japan) at 420 nm.

#### 2.3.3. Circular Dichroism (CD) Analysis

CD spectra of the PSP and PSP−Zn (2.0 mg/mL) were obtained on a Chirascan CD spectropolarimeter (Applied Photophysics Ltd., Surrey, UK) over the wavelength range of 190–260 nm at a scan rate of 100 nm/min and a bandwidth of 2.0 mm.

#### 2.3.4. Fourier Transform Infrared (FT-IR) and Ultraviolet–Visible (UV-Vis) Analysis

The FT-IR of PSP and PSP−Zn were obtained on a Nicolet iS10 FT-IR spectrometer (Thermo Fisher Scientific co., Ltd., Waltham, MA, USA) over the range of 400–4000 cm^−1^ with 32 scanning times.

The UV-Vis spectra of the PSP and PSP−Zn solutions (2 mg/mL) were obtained on a UV-2450 UV-Vis spectrometer (Shimadzu, Japan) over the wavelength range of 200–500 nm.

#### 2.3.5. Morphological Analysis

The surface morphologies of PSP and PSP−Zn were observed under a SUPRATM 55 scanning electron microscope (Carl Zeiss AG, Oberkochen, Germany). The samples were fixed onto the SEM stage and sprayed with gold powder. Images of each sample were acquired at 200× and 5000× magnification.

#### 2.3.6. Particle Size, Polydispersity Index (PDI), and Zeta Potential

The particle size, the polydispersity index, and zeta potential of PSP and PSP−Zn (1.0 mg/mL) were measured using a Zetasizer Nano ZS90 analyzer (Malvern, Malvern Town, UK) at 25 °C. The calculation of particle size and zeta potential were based on the Stokes–Einstein equation and Smoluchowski model, respectively.

#### 2.3.7. X-ray Diffraction (XRD)

The XRD diffractograms of PSP and PSP−Zn were obtained on an EMPYREAN diffractometer (Panaco, Netherlands) at an angle of 2θ, from 5° to 80° at a scanning rate of 5°/min.

#### 2.3.8. Thermogravimetric Analysis (TGA)

The TGAs of PSP and PSP−Zn were performed using an STA6000 thermogravimetric analyzer (PerkinElmer, Waltham, MA, USA). Equal masses of the samples (3.0 ± 0.1 mg) were placed in an alumina crucible and heated from 50 to 600 °C at a heating rate of 10 °C /min.

### 2.4. Stability Evaluation

Equal volumes of the PSP and PSP−Zn samples (1.0 mg/mL) were placed under the following conditions: different temperatures (40, 50, 60, 70, and 80 °C) for 1 h, different time periods (7, 14, 21, 28 D) at 4 °C, and pH-adjusted solutions (2.0, 4.0, 6.0, 8.0 and 10.0) at 4 °C for 24 h. The particle size, PDI, and zeta potential were measured using a Zetasizer Nano ZS90 analyzer, as described in Section 2.3.6.

### 2.5. Anti-inflammatory Activity of PSP and PSP−Zn

#### 2.5.1. Zebrafish Maintenance

Adult Zebrafish (Tg:zlyz-EGFP) were administered at 28 °C with a 14 h light/10 h dark cycle in a 3 L acrylic tank. Zebrafish were fed three times per day. The day before mating, zebrafish were randomly selected for interbreeding in a female:male ratio of 2:2. The naturally spawned embryos were collected within 30 min by switching on the lights in the morning.

#### 2.5.2. Effects of PSP and PSP−Zn on Macrophages and Inflammatory Factors in Zebrafish

Three days post-fertilization (3 dpf) hatched larvae were transferred into 24-well plates, 12 larvae for each cell. Then, the larvae were treated with different concentrations (5, 10, 50, 100, 200 µg/mL) of PSP and PSP−Zn for 3 h. Embryo medium was used as the negative control, and ibuprofen (20 µM) as the positive control. After the treatments, the larvae were exposed to CuSO4 (40 µM) for another 2 h. An SZX16 fluorescence microscope (Olympus, Japan) was used to obtain the photographs of fluorescent neutrophils and macrophages. The migration number of neutrophils was counted by Image pro-plus software.

#### 2.5.3. RNA Extraction and Real-time Quantitative Polymerase Chain Reaction (qPCR)

3 dpf larvae were treated with or without PSP and PSP−Zn for 12 h, as described in Section 2.5.2. Larvae in the same group were collected and total RNA was extracted using an RC101 RNA extraction kit (Vazyme, Nanjing, China). After that, cDNA was generated using the HiScript ^®^III RT SurperMix for qPCR (+gDNA wiper) kit following the manufacturer’s instructions. The resulting cDNA was used to perform qPCR using the 2×ChamQ Universal SYBR qPCR Master Reagent kit. The specific primer pairs used in this study are shown in Table 1.

### 2.6. Statistical Analysis

All experiments were conducted in triplicate, with results expressed as the mean ± standard deviation and evaluated using a one-way analysis of variance (ANOVA). Statistical analysis was performed using the SPSS software (version 26.0, Armonk, NY, USA), and *p*-values < 0.05 were considered statistically significant by Tukey’s test.

## 3. Results

### 3.1. Structural Characterization of PSP−Zn

The type of monosaccharide and molecular size are pivotal in the biological activity of polysaccharides [18]. To clarify the structural characterization properties and the complex formation mechanism of PSP−Zn complexes, the molecular weight, monosaccharide composition, particle size, secondary structure, functional groups, crystal structure, morphological analysis, and thermogravimetric analysis of PSP and PSP−Zn were all evaluated.

#### 3.1.1. Molecular Weight (Mw) and Monosaccharide Composition

As shown in Table 2, there were some differences in the molecular weight and monosaccharide composition of PSP and PSP−Zn. According to the regression curve equation of dextran standards, the Mw values of PSP and PSP−Zn were approximately 3.034 × 10^6^ Da and 3.222 × 10^6^ Da, respectively. The polydispersity coefficient of Mw changed from 4.02 to 4.2 after the chemical modification of PSP. In general, the relative molecular weight of PSP increased slightly as polymer diameter increased after the chemical modification by Zn^2+^. Zhang et al. [22] also reported that after zincification, the molecular weight of *Fritillaria ussuriensis* polysaccharide increased from 1.56 × 10^6^ to 1.86 × 10^6^ Da.

The monosaccharide composition result showed that PSP was mainly composed of rhamnose, arabinose, galactose, glucose, and galacturonic acid. The PSP−Zn complex exhibited a similar monosaccharide composition to PSP except for the molar ratio of monosaccharides. Specifically, the monosaccharide molar ratio of PSP was 2.17:3.63:7.38:75.04:11.78, and that of PSP−Zn was 1.96:3.01:6.81:81.54:6.68. The decrease in galacturonic acid in the PSP−Zn complex suggested that Zn^2+^ mainly interacted with the galacturonic acid residues of chains. Galacturonic acid possesses strong affinity to bind with metal ions owing to its negatively charged carboxylic groups [23,24].

#### 3.1.2. Circular Dichroism (CD) Analysis

**Figure 1:**
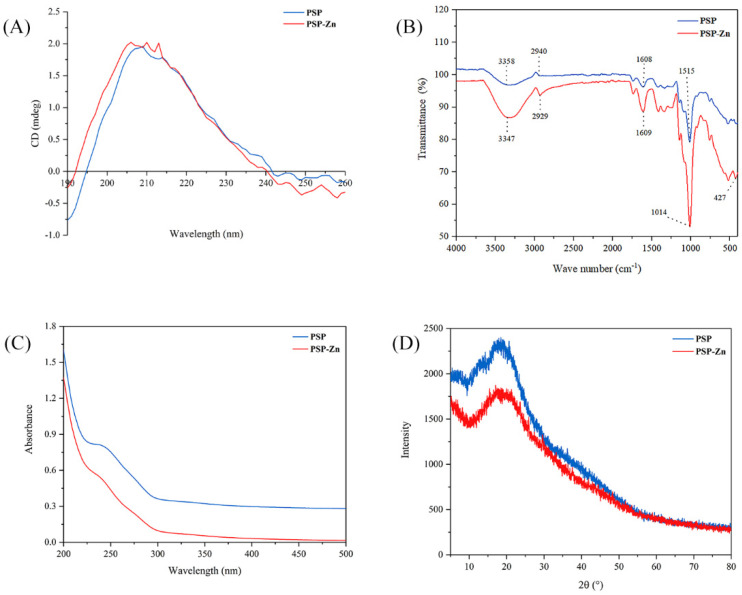
(**A**) CD spectrogram, (**B**) FT-IR spectrogram, (**C**) UV–visible spectroscopy, and (**D**) XRD patterns of PSP and PSP−Zn.

Circular dichroism is a common method used to study the secondary structure of polysaccharides. The spectra exhibited a broad positive peak ranging from around 190 to 245 nm (Figure 1A). PSP has a strong absorption peak in the far ultraviolet region, especially at 190 nm. PSP and PSP−Zn had positive and negative cotton effects at the wavelength range of 190–260 nm. The absorption peak of PSP−Zn at 209 nm was slightly enhanced and a new absorption peak at 206 nm was observed, which showed higher asymmetry. The results indicated that the chelation occurred through Zn^2+^ binding with the hydrogen bond in the polysaccharides, which caused a change in the conformation of the molecule [25]. This result is similar to the research results of Zhang et al. [26].

#### 3.1.3. Fourier Transform Infrared (FT-IR) Spectrum Analysis

The FT-IR spectrum of PSP and PSP−Zn complex are depicted in Figure 1B. As shown in the figure, a broad peak appeared at 3358 cm^−1^, belonging to the O–H stretching vibration, owing to the fact that intramolecular or intermolecular hydrogen bounds are easily formed in PSP. The peak became sharp and strong after modification with Zn^2+^, which was also observed in *Fructus Mori* polysaccharide–Zn complex [27]. The absorption peaks at 2940 and 1608 cm^−1^ were related to C–H stretching vibration and C=O stretching vibration, respectively [28]. Additionally, the absorption bands in the range of 980–1250 cm^−1^ are ascribed to the vibrations of pyranoid rings. In comparison to PSP, considerable changes were observed in the main characteristic absorption bands. Most of the PSP−Zn absorption peaks were more significant and shifted toward lower wavenumbers at varying degrees, suggesting that the Zn^2+^ ions had successfully coordinated with the PSP. Notably, there was a new absorption peak at 427.66 cm^−1^, belonging to Zn–O, further indicating that Zn^2+^ had coordinated with the PSP.

#### 3.1.4. Ultraviolet–Visible (UV-Vis) Spectrum Analysis

As shown in Figure 1C, PSP and PSP−Zn exhibited invisible absorption peaks at 280 nm, but the absorption intensity of PSP was significantly greater than that of PSP−Zn. This result suggests that a small amount of protein or polypeptide was carried in PSP and PSP−Zn [29]. The reduction in absorption intensity was due to the fact that the bonding effect between zinc ion and hydroxyl resulted in a reduction in the conjugated system [22].

#### 3.1.5. X-ray Diffraction (XRD) Analysis

Crystalline features have a vital impact on various physicochemical properties of a substance, such as solubility, hydrolysis, swelling, and viscosity, which can limit its application. The X-ray diffraction (XRD) patterns of PSP and PSP−Zn are shown in Figure 1D. There was only one broad diffraction peak at 20°, depicting the amorphous nature of PSP and PSP−Zn. There was no crystalline diffraction peak corresponding to ZnSO_4_, suggesting that the Zn^2+^ ions were completely used to form the PSP−Zn complex. A similar result was reported by Wang et al. [2]: that zinc–Hohenbuehelia serotina polysaccharide complexes possessed a semi-crystalline nature. However, the chitosan–zinc and amylase–zinc complexes only showed partial crystalline properties [30,31]. The reason might be due to the fact that the chitosan and amylase were composed of homogeneous class units and ordered structure, whilst PSP is a heterogeneous class of carbohydrate polymers. The chemical modification with zinc did not rearrange the polysaccharide molecular structure [2].

#### 3.1.6. Thermogravimetric Analysis (TGA)

**Figure 2:**
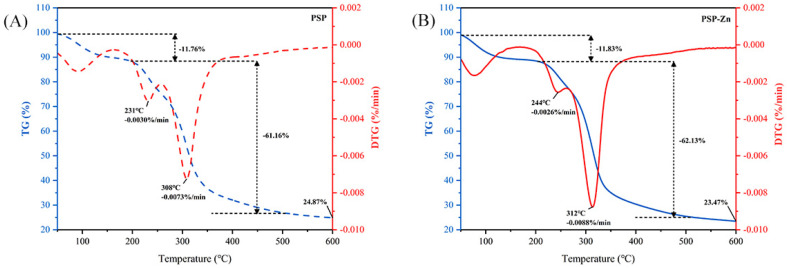
Thermogravimetric curve of (**A**) PSP and (**B**) PSP−Zn.

TGA was used to investigate the thermal stability and decomposition pattern of PSP and PSP−Zn. As presented in Figure 2, PSP exhibited a reduction in mass of 11.76% in the range of 50–204 °C, which might be related to the loss of free water. A sharp decrease in PSP mass occurred in the range of 204–348 °C. The maximum mass loss rate was 0.0073%/min at 308 °C, decreasing above 308 °C, which indicated that the PSP decomposed and its structure was destroyed. PSP−Zn reduction in mass of 11.83% from 50 °C to 219 °C might correspond to the sublimation of adsorbed water. In the range of 219–334 °C, PSP−Zn underwent violent thermal decomposition, and the mass loss rate reached up to 0.0088%/min at 312 ºC. When the temperature was above 512 °C, the mass loss rate of PSP−Zn leveled off, and the final residual mass was about 23.47% of the original mass. The above results show that the thermal stability of PSP decreased slightly after chelating with Zn^2+^ in the heating temperature range, which is in agreement with previous report by Zhang et al. [26].

#### 3.1.7. Morphological Analysis

**Figure 3:**
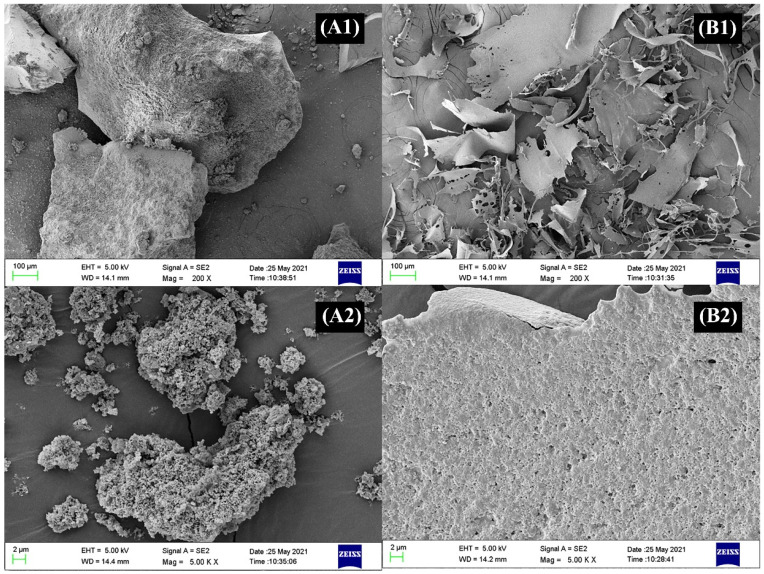
SEM images of (**A1**,**A2**) PSP and (**B1**,**B2**) PSP−Zn.

The morphological changes of polysaccharides before and after metal ion modification were observed by the scanning electron microscope (SEM). As shown in Figure 3, the morphologies of PSP exhibited a block structure with rough surface and porous features. The morphology of PSP−Zn complex was substantially different from that of PSP. The PSP−Zn complex exhibited lamellar texture with a smooth surface and diminished holes, which suggested that Zn^2+^ was successfully bonded onto the PSP. Moreover, the intermolecular cross-linking increased as Zn^2+^ coordinated with the PSP molecules, and the conformational changed. The above results are consistent with the results of PSP and PSP−Zn complex particle size.

#### 3.1.8. Particle Size Analysis

**Figure 4:**
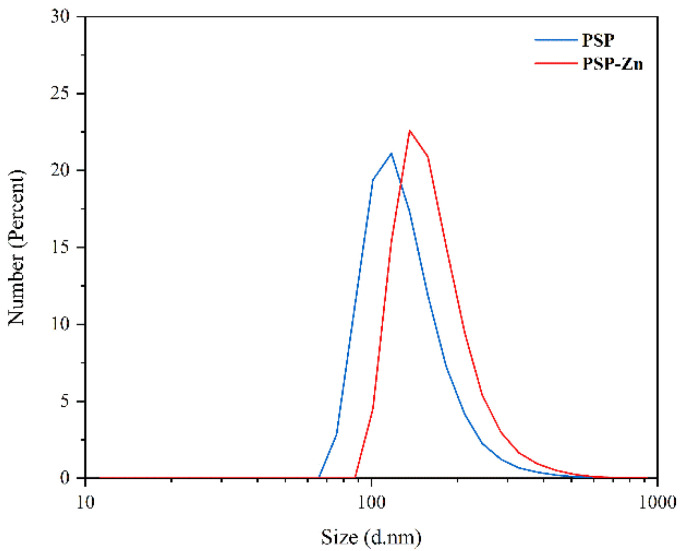
The particle size of PSP and PSP−Zn.

The particle size of PSP and PSP−Zn are shown in Figure 4. The particle size ranges of PSP and PSP−Zn were 97.6–117.6 nm and 136.2–157.2 nm, respectively. In comparison to PSP, the particle size of PSP−Zn increased, which may be due to the fact that during the complexation process, the polysaccharides chains opened, and zinc ions binding to the hydroxyl group on the polysaccharide chains caused aggregations.

### 3.2. Stability Evaluation

PDI was used to measure the size distribution of polysaccharide particles in solutions. Smaller PDI corresponds to narrower particle size, which indicates the that the polysaccharide solutions had good homogeneity [32]. PDI > 0.7 indicates a wide particle size distribution [33]. Zeta potential was used to measure the strength of mutual attraction and the repulsion of particles in water; a higher zeta potential indicated more a stable polysaccharide solution [34].

**Figure 5:**
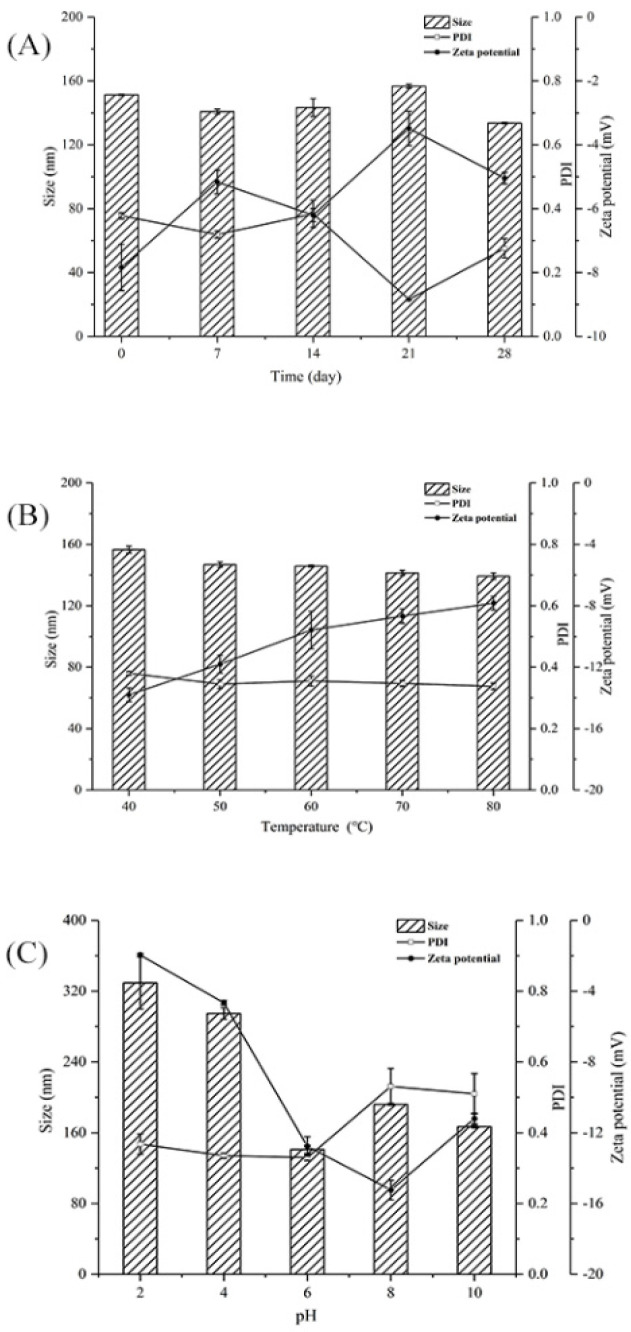
(**A**) Storage, (**B**) thermal, and (**C**) pH stability of PSP−Zn solutions.

Figure 5A shows the effect of storage time on the particle size, PDI, and zeta potential of PSP−Zn at 4 °C. The particle size, PDI, and zeta potential absolute values of PSP−Zn all decreased, which indicated that the dispersibility and stability of PSP−Zn decreased as storage time was prolonged. It was speculated that the modification with Zn^2+^ reduced the repulsive force between molecules, which easily caused the aggregation of the polysaccharides and resulted in decreased stability [35]. Figure 5B shows the effect of temperature on the stability of PSP−Zn: the particle size decreased from 156.5 nm to 139.33 nm, PDI was about 0.3, and the absolute value of zeta potential decreased from 13.8 to 7.83 as temperature increased from 40 to 80 °C. This result indicates that the stability decreased with the increase in temperature; it is speculated that high temperature could destroy the structures of polysaccharides and weaken the surface charge between PSP and Zn^2+^, leading to the lower stability of the solution system [36]. Figure 5C shows the effect of different acidic and alkaline environments on the stability of PSP−Zn: the particle size of PSP−Zn increased significantly, PDI > 0.7, and the absolute value of zeta potential was relatively small (2–4) under acidic conditions (pH 2 and 4), indicating a highly unstable state. It was speculated that due to the protonation of polysaccharides under strongly acidic conditions, the electrostatic interaction between PSP and Zn^2+^ was weakened, leading to the aggregation of the samples and the increase in particle size. In contrast, PSP−Zn showed a small change in particle size under weak acid (pH 6) and alkaline conditions (pH 8 and 10), in which PDI was 0.3–0.5 and the absolute value of zeta potential increased (11–15), indicating a stable state. These results are consistent with a previous report by Tang et al. (2020) [37]. However, Qiu et al. reported that nanoparticle consisting of pectin were more stable under acidic conditions [38]. The reason might be due to the fact that different ratios of COO- groups in biomolecules lead to differences in pH sensitivity.

### 3.3. Anti-inflammatory Activity

#### 3.3.1. Effects of PSP and PSP−Zn on the Movement of Inflammatory Cells in Zebrafish

**Figure 6:**
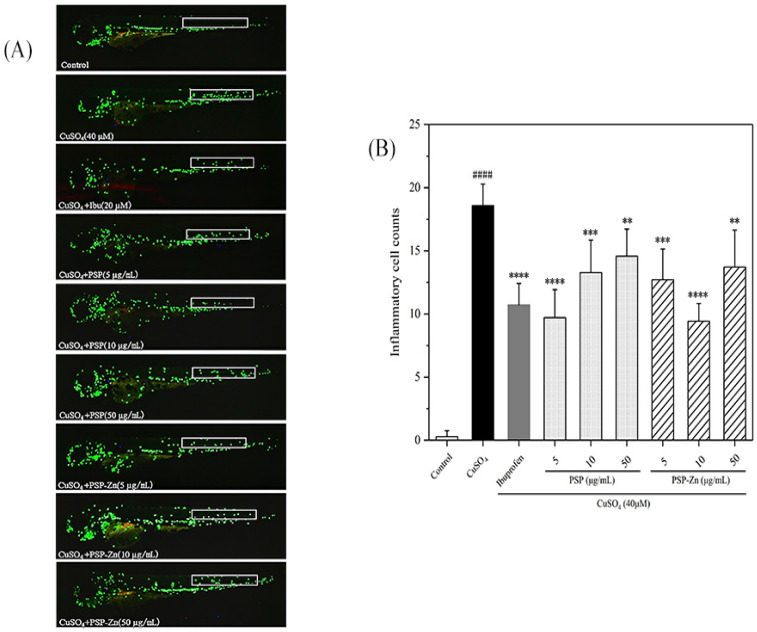
Anti-inflammatory activity of PSP and PSP−Zn in CuSO_4_-induced larvae. (**A**) In vivo representative fluorescence images of zebrafish macrophage migration. (**B**) Statistical results of the number of macrophage migrating in zebrafish lateral line (white square). Data are shown as mean ± SD with n = 7. #### *p* < 0.0001 as compared with the control group; ** *p* < 0.01, *** *p* < 0.001, and **** *p* < 0.0001 as compared with the model group.

Zebrafish, one of the vertebrate models, have 87% homology with humans and are widely used in the analysis of human inflammation [39]. As shown in Figure 6A, in the model groups, a large number of macrophages were observed at the zebrafish lateral line, indicating that the inflammation model had been established successfully. In a preliminary experiment, we first investigated the anti-inflammation ability of different concentrations of PSP and PSP−Zn; the results showed that PSP and PSP−Zn above 100 µg/mL had limited effect on macrophage migration. Therefore, PSP and PSP−Zn at 5, 10, 50 µg/mL were used in the following experiments. As shown in Figure 6B, compared with the model groups, ibuprofen (Ibu) inhibited the recruitment of macrophages (*p* < 0.0001) and the cell migration rate decreased by 42.31%, which indicated that Ibu has a good inhibitory effect on the inflammatory response. PSP and PSP−Zn dose-dependently inhibited the migration of macrophages. For PSP and PSP−Zn at the concentrations of 5, 10, 50 µg/mL, the macrophage migration rate decreased by 47.69%, 28.46%, 21.54% and 31.54%, 49.23%, 26.15 %, respectively. PSP at 5 µg/mL and PSP−Zn at 10 µg/mL inhibited more recruitment of macrophages than 20 µM Ibu; however, there was no significant difference (*p* ≥ 0.05) among them. This result indicates that PSP and PSP−Zn also have a good inhibitory effect on inflammation; more precisely, 5 µg/mL PSP and 10 µg/mL PSP−Zn were optimal concentrations for anti-inflammation.

#### 3.3.2. Effects of PSP and PSP−Zn on the Expression of Inflammatory Factors in Zebrafish

To preliminarily explore the mechanisms underlying the anti-inflammatory effects of PSP and PSP−Zn, RT-PCR was used to determine the transcription factors and pro-inflammatory cytokines of the NF-κB pathway. NF-κB is an important factor in the inflammatory response pathway, and activation of the signaling pathway will produce a large number of downstream inflammatory factors. The cascade of NO increases and amplifies the inflammatory response. iNOS is the rate-limiting enzyme of NO. In the event of inflammation, iNOS can stimulate NO production in body tissues and cells, which brings inflammatory damage to the body [40].

**Figure 7:**
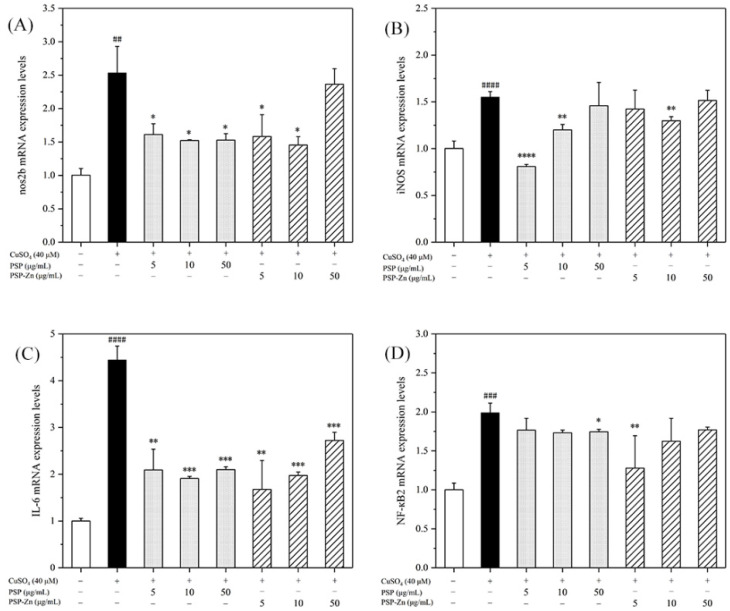
RT-PCR detects the expression of (**A**) nos2b, (**B**) iNOS, (**C**) IL-6, and (**D**) NF-κB2. Data are shown as mean ± SD with n = 3. ## *p* < 0.01, ### *p* < 0.001, #### *p* < 0.0001 versus the control group; * *p* < 0.05, ** *p* <0.01, *** *p* < 0.001, and **** *p* < 0.0001 versus the model group.

As shown in Figure 7, the mRNA expression levels of nos2b, iNOS, IL-6, and NF-κB2 in the model group increased by 1.5- to 4-fold compared with that of the control group. After being treated with PSP and PSP−Zn, the mRNA expression levels of pro-inflammatory cytokines decreased by varying degrees compared with that of the model group. These results indicate that PSP and PSP−Zn exert anti-inflammatory effects in CuSO_4_-stimulated zebrafish. As described by Li et al. [5], biological polysaccharides lead cells that have been exposed to inflammation to attenuate the secretion of pro-inflammatory cytokines (IL-6), inflammatory mediator (iNOS), and toxic molecules (NO). Meanwhile, Feng et al. [41] revealed that zinc supplements could also attenuate the level of pro-inflammatory cytokines. Effective control these inflammatory factors would protect cells from inflammation damage and apoptosis [42]. Taken together, it was speculated that the anti-inflammatory mechanism of PSP and PSP−Zn were through the downregulation of CuSO4-stimulated NF-κB pathway activation, which effectively inhibited NO production [43,44].

## 4. Conclusions

In the present work, polysaccharides from pumpkin skin were extracted and purified. A novel PSP−Zn complex was successfully prepared, and the physicochemical, structural characteristics, and in vivo anti-inflammation of PSP−Zn were evaluated, respectively. The results indicated that a novel PSP−Zn complex was successfully prepared, and zinc ions might chelate through hydroxyl groups. The PSP−Zn complex was a semi-crystalline substance with lamellar texture and smooth surface morphology, possessing higher stability in weak acid and alkaline environments. PSP did not change its anti-inflammatory activity after conjunction with zinc. In vivo, PSP and PSP−Zn downregulated the expression of nos2b, iNOS, IL-6, and NF-κB2 in zebrafish larvae induced by CuSO_4_, which suggested that PSP and PSP−Zn exhibited anti-inflammatory activity. These results indicate that PSP−Zn could potentially be used as an effective and safe form of zinc supplementation.

## Figures and Tables

**Table 1:** The specific primer pairs used in this study.

Name	Primer Orientation	Forward and Reverse Primer Sequences (5′-3′)
β-actin	Forward	CTCCGGTATGTGCAAAGC
	Reverse	CCATCACTCCCTGATGTCT
IL-6	Forward	ACGACATCAAACACAGCACC
	Reverse	TCGATCATCACGCTGGAGAA
NF-κB2	Forward	ACAAGACGCAAGGAGCCCAG-
	Reverse	AACTGTCTCTTGCACAAAGGGCTCA
nos2b	Forward	ACTTTCGGCTGCTTTTCTTCT
	Reverse	GGACCTTTTCCCTCCTGTGTA
iNOS	Forward	GGAGATGCAAGGTCAGCTTC
	Reverse	GGCAAAGCTCAGTGACTTCC

**Table 2:** The molecular weight and monosaccharide composition of PSP and PSP−Zn.

Parameters		PSP	PSP−Zn Complex
Molecular weight (Da)		3.034 × 10^6^	3.222 × 10^6^
Polydispersity coefficient		4.02	4.2
Monosaccharide composition (%)	Rhamnose	2.17	1.96
	Arabinose	3.63	3.01
	Galactose	7.38	6.81
	Glucose	75.04	81.54
	Galacturonic acid	11.78	6.68

## Data Availability

Data is contained within the article.
